# Editorial: Brain Mechanisms Linking Sleep and Epilepsy

**DOI:** 10.3389/fnhum.2022.922372

**Published:** 2022-05-10

**Authors:** Vasileios Kokkinos, Andreas M. Koupparis, Michalis Koutroumanidis, George K. Kostopoulos

**Affiliations:** ^1^Department of Neurosurgery, Massachusetts General Hospital, Boston, MA, United States; ^2^Harvard Medical School, Boston, MA, United States; ^3^Institute of Neurology and Genetics, Nicosia, Cyprus; ^4^Department of Clinical Neurophysiology and Epilepsies, Guy's and St. Thomas' NHS Foundation Trust, St. Thomas' Hospital, London, United Kingdom; ^5^Institute of Psychiatry, Psychology and Neuroscience, King's College London, London, United Kingdom; ^6^Neurophysiology Unit, Department of Physiology, Medical School, University of Patras, Patras, Greece

**Keywords:** epilepsy, sleep, sleep-related epilepsies, nocturnal seizures, neurophysiology

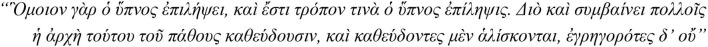

“*Hence too they are liable to epilepsy, for sleep is like epilepsy; indeed in a sense sleep is an epileptic fit. Consequently for many people epilepsy begins in sleep, and they are regularly seized with it when asleep, but not when awake”*

Aristotle, De somno et vigilia, 4^th^ century BCE

The strong link between epileptic seizures and sleep was first suggested by Aristotle, who associated the higher prevalence of epilepsy with the larger amount of sleep in childhood and observed that some people had seizures while asleep but not when awake (Temkin, 1994). The clinical observations by Gowers and Féré were duly followed only by the end of the nineteenth century (Gowers, 1881; Féré, 1890), and thereby today we know of a number of epileptic syndromes and epilepsies, the seizures and EEG epileptic activity of which show distinctive intimate associations with sleep. These sleep-related epilepsies (SRE) do not form a homogenous group. They affect children and adolescents/adults alike, manifest with focal or generalized seizures or both and are of structural or genetic etiologies (Nobili et al., 2020). Furthermore, clinical-EEG studies over the last five decades or so have shown that the interface between these SRE and sleep is far from uniform. In the self-limited focal epilepsies of childhood, such as Rolandic epilepsy and Panayiotopoulos syndrome, focal seizures and interictal epileptic discharges increase or only occur during sleep (Panayiotopoulos et al., 2008), while seizures in sleep-related hypermotor epilepsy occur almost exclusively from sleep (Tinuper et al., 2016). On the other hand and at variance with this “all in sleep” pattern, genetic (idiopathic) generalized epilepsies show a relative electroclinical dissociation; while absences, myoclonic and generalized tonic-clonic seizures are known (since the original description by Janz) to mainly occur on awakening (Janz, 1953, 2000) and also during the waking state, generalized spike-wave discharges increase during all stages of slow sleep when seizures occur only rarely.

Dynamic interactions between SRE and sleep are reciprocal, introducing a vicious cycle: nocturnal epileptic seizures disrupt the so much needed restorative slow sleep, compromising seizure control through poor sleep quality and deprivation; this, in turn, incurs more seizures. The stability of the sleep microstructure is also affected by interictal epileptic discharges, mainly generalized (Gigli et al., 1992), which may also impact on seizure control (Serafini et al., 2013). Last but not least, increased epileptic EEG activity in sleep (with or without seizures) affects high cognitive functions with the Encephalopathy with Status Epilepticus during Sleep and Landau-Kleffner syndrome being primary examples (Tassinari et al., 2009), while cognition in self-limited focal epilepsies of childhood is under intense investigation (Pal et al., 2016).

Experimental evidence increasingly confirm and extend the links between sleep and epilepsy, by demonstrating mechanisms potentially underlying features of both. These mechanisms converge at the activation of the same or similar genes, channels and synapses as well as extensive brain networks. One example is the cortico-thalamo-cortical circuits involved in sleep maintenance (sleep spindles) and loss of consciousness in epileptic absences (3 Hz spike-wave discharges) (Kostopoulos, 2000). Another is the circuits sustaining a cortico-hippocampo-cortical interaction, proposed to be involved in synaptic plasticity-mediated memory processes during slow wave sleep. These processes are characterized by sharp wave ripples, which in mesial temporal lobe epilepsy are compromised by interictal spikes, thus contributing to cognitive deficits (Halász et al., 2019). The multifaceted reciprocal interactions between sleep and SRE of extensive electroclinical and genetic variability, and the interference of epileptic discharges with the plastic brain functions in slow sleep, suggest a constellation of neurobiological mechanisms with complex interactions, which we still know very little about (Kostopoulos, 2009; Halász, 2013; Kostopoulos and Koutroumanidis, 2017).

The goal of this Research Topic was to bring together a variety of neurophysiological perspectives to enhance our understanding of the relationship between sleep and epilepsy. We warmly thank all the contributing authors and reviewers for the precious time and effort they devoted to support this effort. Garg et al., in their systematic review titled “Sleep and temporal lobe epilepsy—associations, mechanisms and treatments” are focusing on the relationship between temporal lobe epilepsy and sleep. They highlight its effects on sleep micro- and macrostructure, quality of sleep, and sleep pathology, and examine the overall effects on cognition and patients' quality of life. Georgopoulou et al., in their review titled “Altered sleep-related consolidation and neurocognitive comorbidity in CECTS” are discussing the effects of sleep-potentiated interictal epileptic activity on cognition in the childhood epilepsy with centro-temporal spikes syndrome and critically investigate the potential pathways through which sleep-related consolidation is distorted by epilepsy. Sitnikova, in the review titled “Sleep disturbances in rats with genetic pre-disposition to spike-wave epilepsy (WAG/Rij)” is presenting evidence from animal models that spike-wave activity disrupts micro- and macro-architecture elements of slow-wave sleep, intermediate sleep stages and microarousals. McLeod et al., in their systematic review and analysis article titled “Can REM sleep localize the epileptogenic zone? A systematic review and analysis” highlight the superior role of REM interictal activity in localizing the seizure onset and epileptogenic zones, and introduce a novel approach for the description of epileptic manifestations between states in the sleep-wake continuum. In turn, Ma et al., in their research article titled “Phase-amplitude coupling and epileptogenic zone localization of frontal epilepsy based on intracranial EEG” are presenting quantitative approaches for the localization of the epileptogenic zone in frontal lobe epilepsy, a focal epileptic syndrome with well documented propensity for sleep-related seizures. Finally, Halász and Szucs, in their hypothesis and theory article titled “Sleep and epilepsy link by plasticity” present extensive research and clinical evidence from published literature to support the original idea that epileptogenesis in the human brain is promoted by plasticity-based transformations of normal neural networks underlying sleep processes. According to this hypothesis, such neurophysiological transformations create a vicious circle of interaction between sleep and epilepsy, affect sleep-consolidated cognition, and are responsible for the high association of epileptic manifestations and seizures with sleep.

Advances in the fields of neurophysiology, neuropsychology, sleep medicine and genetics open up exciting possibilities to improve our understanding on what links sleep and epilepsy, and thereby generate a direct clinical impact. We hope that this Research Topic, jointly hosted by Frontiers in Neurology and Frontiers in Human Neuroscience, provides readers with a comprehensive and useful overview. We further hope that our effort will encourage epileptologists to critically address the sleep/epilepsy relationship and incorporate the current state-of-the-art in their clinical practice.

## Author Contributions

All authors listed have made a substantial, direct, and intellectual contribution to the work and approved it for publication.

## Conflict of Interest

The authors declare that the research was conducted in the absence of any commercial or financial relationships that could be construed as a potential conflict of interest.

## Publisher's Note

All claims expressed in this article are solely those of the authors and do not necessarily represent those of their affiliated organizations, or those of the publisher, the editors and the reviewers. Any product that may be evaluated in this article, or claim that may be made by its manufacturer, is not guaranteed or endorsed by the publisher.
